# Influence of the coagulation bath on the nanostructure of cellulose films regenerated from an ionic liquid solution†

**DOI:** 10.1039/d4ra00971a

**Published:** 2024-04-22

**Authors:** Lassi V. Tiihonen, Gabriel Bernardo, Robert Dalgliesh, Adélio Mendes, Steven R. Parnell

**Affiliations:** a Faculty of Applied Sciences, Delft University of Technology 2629 JB Delft Netherlands S.R.Parnell@tudelft.nl; b LEPABE – Laboratory for Process Engineering, Environment, Biotechnology and Energy, Faculty of Engineering, University of Porto Rua Dr Roberto Frias 4200-465 Porto Portugal gbernardo@fe.up.pt; c ALiCE – Associate Laboratory in Chemical Engineering, Faculty of Engineering, University of Porto Rua Dr Roberto Frias 4200-465 Porto Portugal; d ISIS Pulsed Neutron and Muon Source, Rutherford Appleton Laboratory Chilton Oxfordshire OX11 0QX UK

## Abstract

Cellulose membranes were prepared from an EMIMAc ionic liquid solution by nonsolvent-induced phase separation (NIPS) in coagulation baths of water–acetone mixtures, ethanol–water mixtures and water at different temperatures. High water volume fractions in the coagulation bath result in a highly reproducible gel-like structure with inhomogeneities observed by small-angle neutron scattering (SANS). A structural transition of cellulose takes place in water–acetone baths at very low water volume fractions, while a higher water bath temperature increases the size of inhomogeneities in the gel-like structure. These findings demonstrate the value of SANS for characterising and understanding the structure of regenerated cellulose films in their wet state. Such insights can improve the engineering and structural tuning of cellulose membranes, either for direct use or as precursors for carbon molecular sieve membranes.

## Abstract 2

The structure of cellulose films prepared by nonsolvent-induced phase separation in coagulation baths of different mixtures and temperatures. High water volume fractions in the coagulation bath result in a highly reproducible gel-like structure with inhomogeneities.

## Graphical abstract

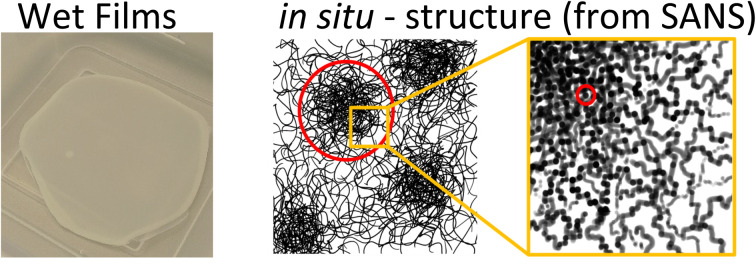

## Introduction

Cellulose is the most abundant renewable biopolymer on earth.^1^ It is found, for example, in a rather pure form in the seed hairs of the cotton plant, or combined with lignin and other polysaccharides (hemicelluloses) in the cell wall of woody plants.^2^ Due to its outstanding properties, such as high biocompatibility, biodegradability and renewability, cellulose has been increasingly used as an alternative to petroleum-based materials for various industrial applications such as paper, packaging and medical products.

Due to its strong inter- and intramolecular hydrogen-bonding network and partially crystalline hierarchical supramolecular structure, cellulose degrades before melting and cannot be melt-extruded into films or fibres. In addition, cellulose does not dissolve in water or in most common organic solvents. Over the last hundred years several non-derivatizing solvent systems have been discovered and utilised, for dissolving cellulose. These include alkaline systems (*e.g.*, sodium hydroxide and lithium hydroxide-based systems), *N*-methylmorpholine-*N*-oxide (NMMO) and ionic liquids.^3–5^

Therefore, cellulose dissolution followed by regeneration in a nonsolvent bath (usually water) is the only viable option for processing the biopolymer without derivatization and shaping it into forms different from its native one. The regeneration process, also known as nonsolvent-induced phase separation (NIPS), of cellulose into flat or hollow-fibre membranes^6,7^ is crucial for several technologically important applications, such as in the preparation of cellulose membranes for liquid^8^ and gas separations^9^ and in the preparation of cellulose precursor films for carbon molecular sieve membranes for gas separation.^10–12^

**Fig. 1:**
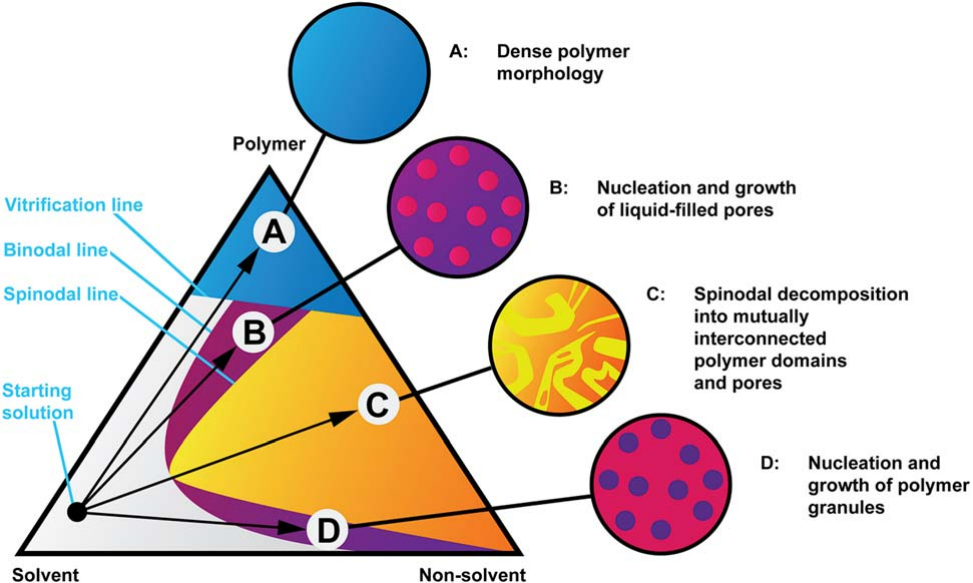
Isothermal phase diagram of a hypothetical polymer, solvent and non-solvent system showing four coagulation routes and representations of the resulting morphologies. Readapted from ref. 32.

Structure formation during solution processing from highly concentrated cellulose solution dopes is a crucial step in controlling the final membrane properties, be they hollow fibres or flat membranes. Cellulose membranes are usually regenerated in water at room temperature. During the regeneration process, the solvent diffuses from the cast film to the water bath (solvent outflow) and water diffuses from the coagulation bath into the cast film (nonsolvent inflow). This solvent–nonsolvent liquid exchange moves the solution composition inside the polymer film from the thermodynamically stable one-phase region to the metastable or unstable two-phase region, originating de-mixing into two phases: a polymer-rich phase that, after solidification, converts into the matrix of membranes and secondly a polymer-poor phase that originates the membrane pores after removal of the solvent and nonsolvent. The final pore structure of the coagulated cellulose membrane is dictated by a complex interplay between thermodynamic factors, as the ternary phase diagram in Fig. 1 exemplifies and kinetic factors, such as the solvent/nonsolvent exchange rate and the kinetics of the de-mixing process. These thermodynamic and kinetics factors are themselves dictated by the physical and chemical properties of the polymer, solvent and nonsolvent and by the binary interactions of solvent–nonsolvent, polymer–solvent and polymer–nonsolvent. Other factors such as the polymer concentration in solution also play a role. Although water is commonly used as a nonsolvent for cellulose regeneration, other nonsolvents such as alcohols can also be used such as in ref. 13. Little is currently known on the structure formation process at the nanoscale, which is not surprising in view of multiple phenomena occurring simultaneously. Previous studies have pointed towards hydrogen bonding being a leading mechanism behind structure formation^14,15^ while this notion has also been criticised.^16^

Several studies have previously addressed the impact of coagulation conditions on the structure formation of cellulose membranes processed from: (i) aqueous alkaline NaOH^17–25^ and LiOH^26–30^ solutions, (ii) NMMO solutions^31–40^ and (iii) ionic liquid solutions.^39,41–47^ Among the coagulation conditions considered were: (i) cellulose molecular mass;^31^ (ii) cellulose concentration in solution;^26,30,31,33,34,36^ (iii) chemical nature of cellulose solvent;^34,37,39,43,45^ (iv) chemical nature of coagulation bath;^17–19,22,23,25,27–29,31,33,36,38,41–44^ (v) coagulation bath temperature^20,21,24–26,33,36^ and (vi) coagulation bath viscosity.^40^

The increase in the coagulation bath temperature has been shown to increase the size of nanometer pores in cellulose films processed from aqueous alkaline NaOH^20,21,24^ and LiOH^26^ solutions and from NMMO solutions.^33^ This has been attributed to the higher solvent–nonsolvent exchange rate that occurs at higher temperatures. However, no studies are known which investigate the effect of coagulation bath temperature on cellulose films processed from ionic liquids.

Some studies have addressed the chemical nature of coagulation baths on the structural formation of cellulose membranes processed from ionic liquids.^41–44^ Durmaz *et al.*^43^ regenerated cellulose membranes from 1-ethyl-3-methylimidazolium acetate (EMIMAc) and from EMIMAc : DMSO solutions, using ethanol and water as coagulants. The phase inversion rate was slower in ethanol, which was attributed to the lower diffusivity of ethanol compared to water. After drying, membranes performed equally which suggests that the microporous structure collapses to a similarly dense structure upon drying. SEM showed that changing the nonsolvent (water and ethanol) does not bring an observable difference in the morphology of the membranes. Hedlund *et al.*^44^ regenerated EMIMAc:DMSO based cellulose solutions with concentrations from 5 to 25 wt%, using water and isopropanol as coagulants and used a solvent exchange strategy (water → isopropanol → butanone → cyclohexane) to preserve the generated microstructures upon subsequent drying before analysis. Common observations in both studies^43,44^ are that the crystalline structure changes from cellulose-I to cellulose-II upon regeneration and cellulose regenerated in alcohols (ethanol and isopropanol) has lower crystallinity than cellulose regenerated in water.

Regeneration in different coagulation baths has also been studied from cellulose solutions in ionic liquids other than EMIMAc.^41,42^ Liu *et al.*^41^ studied the regeneration of cellulose dissolved in the ionic liquid 1-butyl-3-methylimidazolium acetate ([Bmim]Ac) in different nonsolvents: compressed CO_2_, water, ethanol and acetonitrile. The morphology of the cellulose films was observed, after drying, using SEM/TEM: films regenerated using water, ethanol and acetonitrile resemble each other but differed from films regenerated in CO_2_. Cellulose dissolved in [TMGH]^2+^[OOCOCH_2_CH_2_OCOO]^2−^/DMSO was regenerated into membranes by Guo *et al.*^42^ using four different baths: ethanol, methanol, 5 wt% NaOH, and 5 wt% H_2_SO_4_ aqueous solutions. SEM analysis was inconclusive. In both studies^41,42^ membranes regenerated in alcohols (ethanol and methanol) also displayed lower crystallinity than cellulose membranes regenerated in aqueous solutions.

In most of the studies reported above, morphological analysis was based on an SEM and TEM study of the dried films. However, this strategy has the drawback that it cannot guarantee that the generated microstructures are preserved upon subsequent drying before electron microscopy analysis. On the other hand, studies of the morphology of regenerated cellulose films in their wet state, *i.e.* before drying, are still very scarce in the literature because these are not amenable to microscopy analysis under high-vacuum.^39^ In the present work, we study the influence of the coagulation bath properties (chemical nature and temperature) on the nanostructure formation of cellulose films processed from an ionic liquid EMIMAc : DMSO solution. Contrary to previous studies, the nanostructure of the regenerated cellulose films was analysed on their coagulated swollen state, *i.e.* before drying, using small angle neutron scattering (SANS). The effect of the chemical nature of the coagulation bath was addressed by testing a large set of coagulation baths with different chemical nature, such as water, methanol, ethanol, acetone as well as water : ethanol mixtures and water : acetone mixtures. The effect of the temperature of the coagulation bath was studied by testing water baths at different temperatures. The results obtained can be used to leverage the development of strategies to tune the porosity of cellulose membranes for different applications.

## Results and discussion

**Fig. 2:**
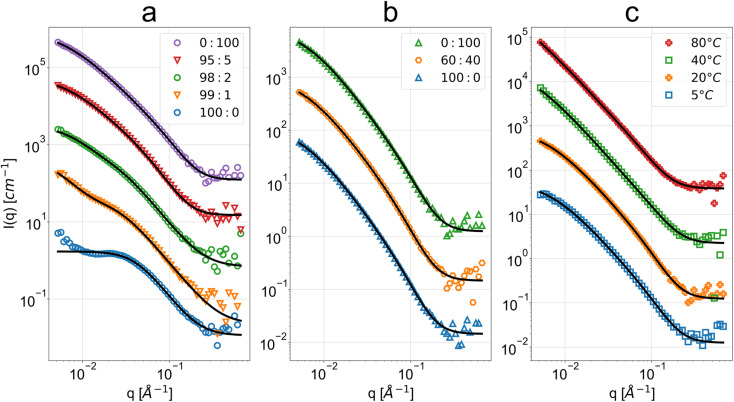
SANS scattering intensity as a function of *q* and respective polymer mass fractal (PMF) or summation of DAB term and Lorentzian term (DL) fits for the regenerated cellulose membranes coagulated in baths at (a) different acetone : H_2_O volume fractions (v/v), (b) different ethanol : H_2_O volume fractions (v/v) and (c) different water bath temperatures. The bottom data sets in blue are at absolute scale, while the subsequent data have been spaced with one decade between them. Some data have been omitted for conciseness due to their high similarity (see ESI for full data and fit results†). Above the high-*q* background level, the error bars of the data points are lower or equal to the size of the markers.

The observed scattering intensities in Fig. 2 reveal high similarity across the three sample sets and their variations, with good description provided by the PMF fit aside from the cases of very high acetone concentrations. This suggests similar cellulose nanostructures across the sample sets. The clear exception is the scattering intensity from the 100 : 0 acetone : H_2_O bath sample, being clearly dissimilar to the rest of the measurements with a weak broad peak around 0.03 Å^−1^. Deviation at a similar *q* is also seen for the 99 : 1 acetone : H_2_O sample. The ethanol–water series shows a similar shape of scattering intensity across the entire bath mixture variations, from pure ethanol to pure water. In the case of water bath temperature effect, for higher temperature water baths (≥40 °C) there is no notable low-*q* flattening of the scattering intensity otherwise observed at lower temperatures and in the other sample sets.

The microbalance method resulted in volume fractions *ϕ*_V_ that are in qualitative agreement with the volume fractions obtained through fitting of SANS results. The mass fractions of cellulose obtained using the microbalance method, and later converted to volume fractions, were highly reproducible for water–acetone mixtures between 80 : 20 and 0 : 100 acetone : H_2_O and all water–ethanol mixtures.

### Acetone–water

**Fig. 3:**
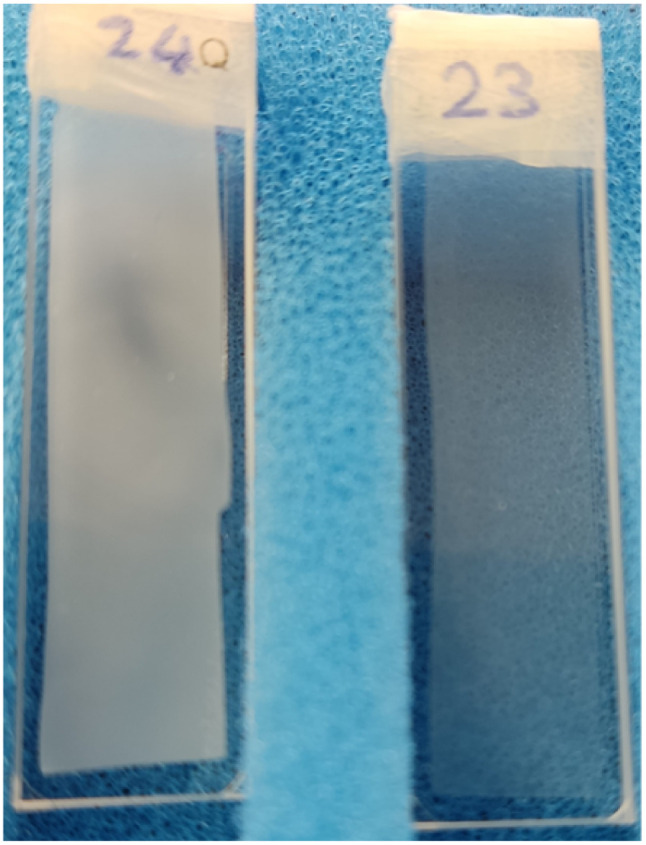
Wet films inside cuvettes with D_2_O. Demonstration of opaqueness difference between a 99.5 : 0.5 (left) and 99 : 1 (right) acetone : H_2_O sample. Higher water fractions resulted in highly transparent film.

The polymer mass fractal (PMF) model describes the data from 95 : 5 to 0 : 100 acetone : H_2_O while the more general summation of a DAB and Lorentzian model term (DL) describes the lower water concentrations down to 99 : 1 acetone : H_2_O (see Table 1). The high-*q* range of 100 : 0 acetone : H_2_O is well-described by the DL model. The low water fraction samples were visually observed to be cloudier and more opaque (see Fig. 3). Calliper measurements of the thickness *t* of the regenerated films showed that these were also significantly thinner (see Table 1).

**Table 1:** Selected acetone : H_2_O sample PMF and DL fit outputs and calliper thickness *t*. (*) diverging parameters. The parameters are described in the Fit models subsection of the Experimental section. The full fit parameters including the omitted fits can be found in ESI

DL ace : H_2_O	*I*_0,*D*_ [10^5^ cm^−1^]	*I*_0,L_ [cm^−1^]	*ξ*_D_ [Å]	*ξ*_L_ [Å]	*d*_L_	PMF ace : H_2_O	*ϕ*_V_	*ζ* [Å]	*d*	*ξ* [Å]	*t* [mm]
100 : 0	0	1.69 ± 0.01	*	24.3 ± 0.1	3.03 ± 0.02	100 : 0	*	*	*	*	0.18 ± 0.02
99 : 1	0.92 ± 0.05	3.2 ± 0.1	175 ± 12	37.3 ± 0.8	2.74 ± 0.03	99 : 1	*	*	*	*	0.12 ± 0.02
98 : 2	2.9 ± 0.1	3.5 ± 0.2	101 ± 3	38.0 ± 0.8	2.77 ± 0.02	98 : 2	*	*	*	*	0.19 ± 0.02
95 : 5	5.5 ± 0.2	3.5 ± 0.2	91 ± 2	36.4 ± 0.9	2.83 ± 0.02	95 : 5	0.26 ± 0.03	169 ± 7	1.94 ± 0.02	14.0 ± 0.4	0.23 ± 0.02
90 : 10	8.3 ± 0.2	4.5 ± 0.2	93 ± 2	36.0 ± 0.8	2.82 ± 0.02	90 : 10	0.18 ± 0.02	171 ± 5	2.03 ± 0.02	12.3 ± 0.3	0.33 ± 0.02
0 : 100	6.8 ± 0.2	3.7 ± 0.2	94 ± 2	36.6 ± 0.9	2.62 ± 0.02	0 : 100	0.13 ± 0.01	171 ± 4	2.07 ± 0.01	8.9 ± 0.3	0.31 ± 0.02

The breakdown of the PMF model at very low water fractions indicates a structural transition, resulting in a very different cellulose nanostructure for a pure acetone bath. The DL fits demonstrate a reduction in the scale factor *I*_0,D_ of the DAB term for larger aggregates at these fractions, indicating the larger structures described by the length scale *ξ*_D_ merge into a well-defined higher-density phase. This phase should have well-defined regular porosity with size on the order of *ξ*_L_ as evidenced by the weak broad peak. However, the high-*q* Porod exponent *d*_L_ of the DL model does not match the *q*^−4^ behavior expected on length scales close to the monomer size, indicating the DL model is not physically representative.

**Fig. 4:**
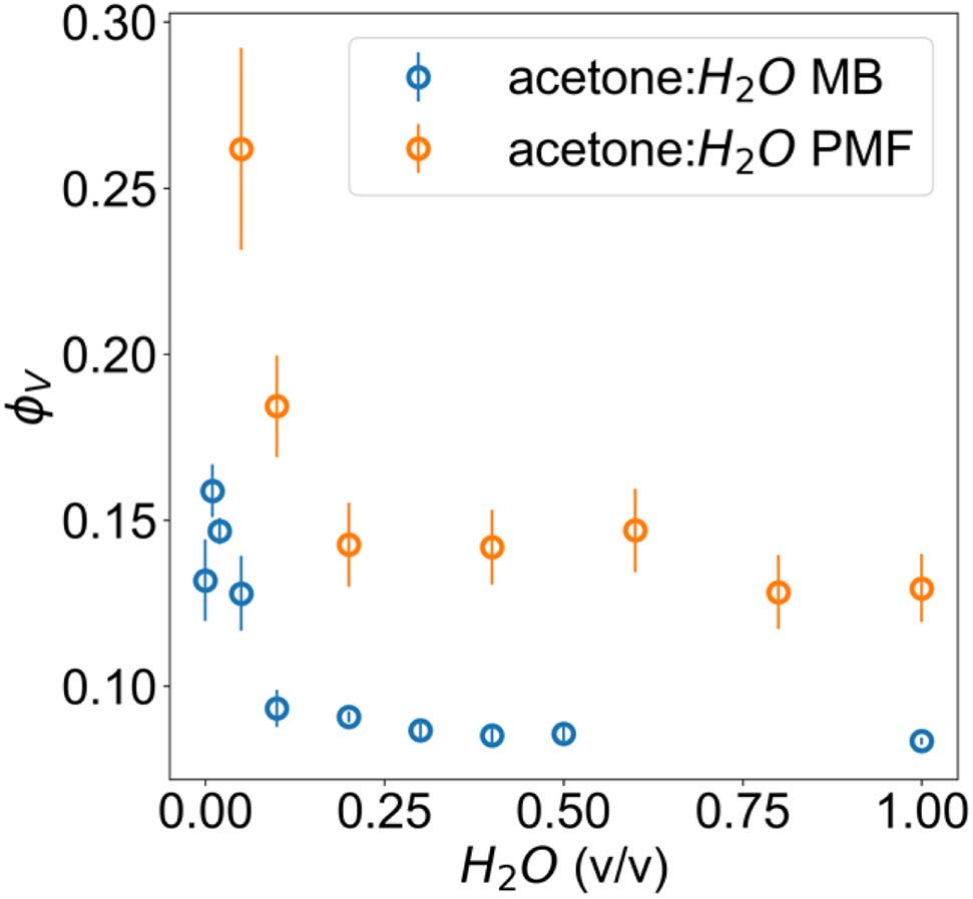
Volume fractions of cellulose in acetone–water membranes measured *via* microbalance (MB) comparison of wet and dry films and estimated from the scattering with PMF fit.

The obtained parameters in the nonsolvent bath range from 95 : 5 to 0 : 100 acetone : H_2_O volume fractions are highly similar, suggesting that above a certain water volume fraction the structure is highly reproducible. This is also supported by the high reproducibility of volume fractions from the microbalance measurements in Fig. 4. The PMF scaling exponent *d* describes the complexity of these aggregates, with the obtained *d* ≈ 2 indicating scattering behaviour similar to ideal chains, while a unit size of *ξ* ≈ 10 Å reasonably matches the diameter of a glucose monomer.

The microbalance measurements show a clear systematic discrepancy against scattering. In the case of scattering, this can be attributed both to the non-spherical form factor of glucose, since the fitting model relies on an assumption of spherical structural units for its scale factor and the possibility of crushing during calliper measurements. These would both result in an upward bias from the true value. For the microbalance method, it is possible that a residual surface film of water was present for the wet film measurement, resulting in an underestimate from the true value.

**Fig. 5:**
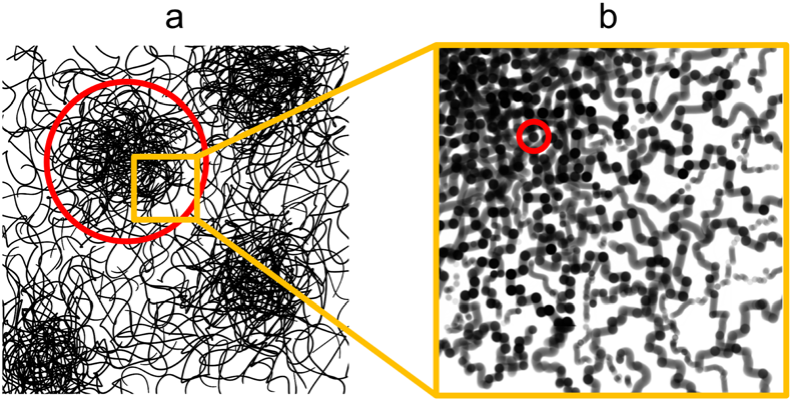
Proposed structure exhibited by water- and ethanol-coagulated cellulose membranes in their wet state with (a) the microstructure consisting of cellulose aggregates with the cutoff length scale indicated in red and (b) the nanostructure of the aggregates with the unit size indicated in red. (a) Adapted from ref. 49 (Fig. 1a).

The pure water structure present from 95 : 5 acetone : H_2_O onwards can be explained as a gel-like structure with local inhomogeneities or polymer-rich phases. This overall structure is described in terms of mass fractal aggregates of cellulose, illustrated in Fig. 5. The prior understanding is that non-solvent induced phase separation should result in well-defined spinodal, enclosed pore or granular structures. In contrast, the indicated structure exhibits granular features but with the distinction that these aggregates are connected and do not have sharply defined interfaces. The porosity of the structure can be described in terms of the size of these clusters given by the mass fractal cutoff length *ζ* and the volume fraction *ϕ*_V_ of cellulose estimated through the scattering and microbalance method. These then also define the size and volume fraction of the polymer-poor phases where the pores originate.

The structure of the film coagulated in pure acetone is interpreted as an asymmetric structure consisting of a very densely packed cellulose layer suggested by the scattering and a macroporous layer that strongly scatters visible light.

Since a small addition of water into the bath results in a structural transition in the cellulose films towards the structure of a film coagulated in pure water, this suggests cellulose interacts preferentially with water over acetone.

### Ethanol–water

The PMF fitting model fully describes the ethanol–water series and the output parameters show high similarity (see Table 2). No significant visual or thickness differences were observed within this series. This suggests no substantial morphological changes within the measured length scale for different ethanol : H_2_O fractions ranging from pure ethanol to pure H_2_O.

**Table 2:** Selected ethanol : H_2_O sample PMF fit outputs and calliper thickness *t*. The parameters are described in the Fit models subsection of the Experimental section. The full fit parameters including the omitted fits can be found in ESI

PMF EtOH : H_2_O	*ϕ*_V_	*ζ* [Å]	*d*	*ξ* [Å]	*t* [mm]
100 : 0	0.13 ± 0.01	188 ± 5	2.09 ± 0.01	10.5 ± 0.3	0.33 ± 0.02
0 : 100	0.13 ± 0.01	171 ± 4	2.07 ± 0.01	8.9 ± 0.3	0.31 ± 0.02

The similarity of the ethanol–water results with pure water suggests that the primary driver may be a chemical interaction. Since water and ethanol have very strong and very weak cohesion respectively, the observations suggest that nonsolvent cohesion plays no critical role in regeneration. However, water and ethanol can both form substantially stronger hydrogen bonds with cellulose than acetone does. This suggests that the presence of strong hydrogen bonders like water and light alcohols with a hydroxyl group results in the hindering of cellulose H-bonding upon solvent exchange by occupying the hydroxyl sites in dissolved cellulose.

Coagulation in pure methanol was also tested (see ESI†) but as the SANS scattering intensities obtained were very similar to pure water and pure ethanol, methanol–water mixtures were not considered.

### Water temperature

The PMF fitting model describes the low temperature samples well, with increasing cutoff length and respective uncertainty from 40 °C onwards (see Table 3). This is consistent with the lack of flattening of the scattering intensity in low-*q*. The high-temperature samples were visually observed to be more opaque. Calliper measurements showed no significant thickness variation between the samples.

**Table 3:** Selected water temperature sample PMF fit outputs and calliper thickness *t*. The parameters are described in the Fit models subsection of the Experimental section. The full fit parameters including the omitted fits can be found in ESI

PMF s H_2_O *T* [°C]	*ϕ*_V_	*ζ* [Å]	*d*	*ξ* [Å]	*t* [mm]
5	0.12 ± 0.01	153 ± 4	2.08 ± 0.01	7.8 ± 0.3	0.27 ± 0.02
20	0.13 ± 0.01	171 ± 4	2.07 ± 0.01	8.9 ± 0.3	0.31 ± 0.02
40	0.09 ± 0.01	268 ± 9	2.12 ± 0.01	5.6 ± 0.3	0.34 ± 0.02
80	0.13 ± 0.01	282 ± 23	2.03 ± 0.01	9.3 ± 0.3	0.34 ± 0.02

The increase in the cutoff length but similarity in other parameters suggests the type of structure remains similar with increasing temperature, but the cutoff length scale of the structure increases. The increasing opaqueness at high temperatures suggests possible microporosity beyond the observation range of SANS, but with the nanostructure otherwise described well as mass fractal aggregates. A likely explanation for both observations is a faster coagulation of cellulose during solvent exchange, resulting in the formation of larger aggregates before the regenerated structure stabilises. The greater parameter uncertainty at higher temperatures can be attributed to an increase in structural variability per sample, while for room temperature regeneration the structure is highly reproducible.

The volume fractions of cellulose in wet films regenerated in water baths at different temperatures were also measured using the microbalance method. The corresponding results (see ESI†) demonstrate that the fraction of cellulose decreases with the increase in the bath temperature. This is in agreement with scattering from the low temperature samples but shows discrepancy for the higher temperatures (≥40 °C), supporting the notion that the true cutoff length scales of the higher temperature samples fall beyond the observable range in SANS.

## Experimental

### Materials

Wood pulp with a degree of polymerization of 450 was provided by Innovia Films Ltd. The ionic liquid 1-ethyl-3-methylimidazolium acetate (EMIMAc) (≥95%), dimethyl sulfoxide (DMSO) (99.9%), pure ethanol, pure acetone and deuterated water (D_2_O) were purchased from Sigma-Aldrich. Ultra-pure water (H_2_O) used in the coagulation bath was obtained from a Millipore water system.

### Fabrication of regenerated cellulose films

Wood pulp (9.2 wt%) was dissolved in EMIMAc:DMSO (30 : 70 wt%) by magnetic stirring for *ca*. 2 h at 90 °C. The EMIMAc : DMSO ratio of 30 : 70 wt% was reported to be ideal for solubilizing cellulose.^10^ The resulting cellulose homogeneous mixture, with a honey brown colour, was degassed by leaving the solution on the hotplate at 90 °C without stirring for *ca.* 10 minutes. Before casting the polymeric precursor film, the mixture was cooled down to 60 °C to increase the viscosity. The polymeric precursor films were spin-coated on square glass plates (10 × 10 cm^2^) with a spin coater (Laurell WS 650). The spin rate used for preparing the cellulose films was 900 rpm with an acceleration of 1000 rpm s^−1^ for 10 s. The glass substrates coated with cellulose film solutions were immersed in water coagulation baths at different temperatures and in chemically different coagulation baths at room temperature (water, ethanol, methanol, acetone, ethanol : water mixtures and acetone : water mixtures). After complete coagulation and detachment of the cellulose films from the glass substrate, the films were rinsed several times in pure water to completely remove the solvents (DMSO and EMIMAc). Then the regenerated cellulose films were rinsed carefully in deuterated water (D_2_O) to exchange H_2_O by D_2_O, and finally they were placed inside quartz cell cuvettes filled with D_2_O. The quartz cells were then placed in the neutron beam for measurement.

### Small-angle neutron scattering (SANS)

Neutron scattering measurements were performed on the Larmor instrument at the ISIS pulsed neutron and muon source. The measurements were carried out in SANS mode. In our case we used neutrons with wavelengths of 0.9–13.5 Å to probe sample structures in the range of 0.1–100 nm based on differences in neutron scattering length density between different phases in the sample. Quartz cell cuvettes filled with D_2_O without a sample were measured to obtain a scattering background and each sample was measured for 45 minutes. The sample structure factors were assumed to be radially isotropic, providing measured intensities per scattering vector magnitude *q*. The data was normalised to absolute units using a polymer reference sample.^48^ The results were analysed by model fitting to obtain structural parameters to study differences between the samples.

### Fit models

The first choice fit model was an established model for the study of gelated polymer systems accounting for static solid-like cross-linked polymer aggregates on the large scale and liquid-like thermodynamic fluctuations on the small scale, summarised by Seiffert:^49^
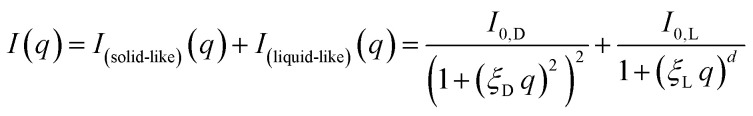


The model abbreviated here as “DL” consists of a Debye–Anderson–Brumberger (DAB) term with a length scale *ξ*_D_ to describe the aggregates and a generalised Lorentzian with a length scale *ξ*_L_ and arbitrary scaling dimension *d* to describe the small-scale fluctuations.

Additionally, a modified mass-fractal model was applied with the base unit form factor being defined by an Ornstein–Zernicke type Lorentzian rather than a sphere as an empirical approximation to the form factor of a glucose molecule:*I*(*q*) = *I*_0_ × *S*(*q*) × *F*^2^(*q*)
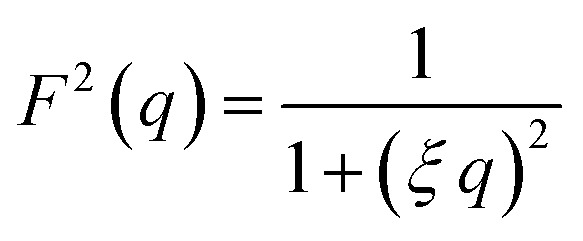

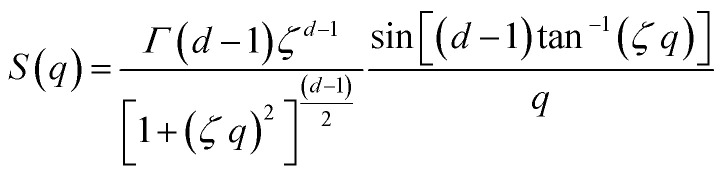


This polymer mass fractal or “PMF” model approximates aggregates of polymers as mass fractals of chained monomers or other relevant structural units. The form factor length scale *ξ* describes the size of the structural unit while the cutoff length *ζ* describes the maximum extent of the mass fractal structures formed by the units, with a scaling dimension *d* related to how compact the structure is. *I*_0_ was later converted to an estimated volume fraction based on the neutron scattering contrast of D_2_O and cellulose based on their respective scattering length densities, estimated at *ρ*_D_2_O_ = 6.4 × 10^−6^ Å^−2^ and *ρ*_cel_ = 1.75 × 10^−6^ Å^−2^ with the SasView SLD calculator and thickness *t* of the wet films as measured with a calliper as:
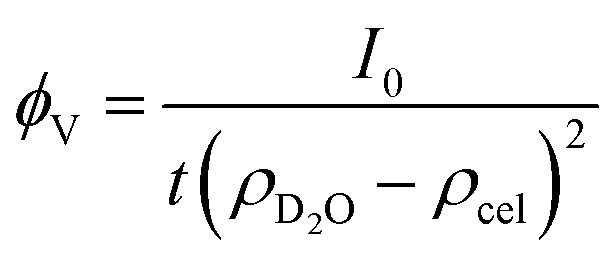
Here a relative error of 3% was assumed for the squared contrast.

The fitting was performed in SasView 5.0.5 with the Levenberg–Marquardt fitting algorithm that uses multidimensional gradient descent to estimate best-fit parameters for the given model and provide error estimates.

### Measurement of polymer percentage using a microbalance

A microbalance was used to determine the mass fraction of polymer in the wet regenerated films, later converted to volume fraction.

The washed wet films were removed from the bath and weighted (*M*_initial_) in an analytical balance with a 10^−4^ g precision. After this they were left drying overnight under ambient air, and then placed in an oven at 40 °C for several hours until they were fully dried (no further mass loss). Finally, they were weighted again (*M*_final_); the mass fraction of cellulose (*ϕ*_m,cel_) in the wet films was calculated by the expression:
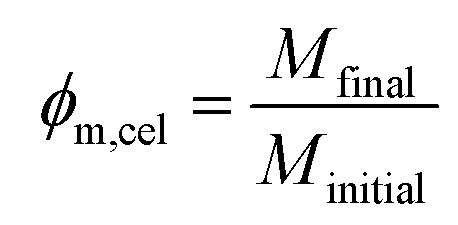


This was converted into a volume fraction of cellulose (*ϕ*_V,cel_) with the following expression with the estimated densities of water (*ρ*_H_2_O_) and amorphous cellulose (*ρ*_cel_):
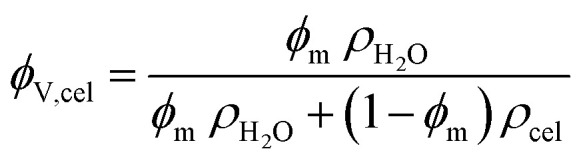


## Conclusions

Wet regenerated cellulose films were studied *in situ* using small angle neutron scattering. Our results show that the properties of the nonsolvent bath used during coagulation can be used to tune the nanostructure of the wet films, offering a new understanding in the field.

An increase in the temperature of the water coagulation bath resulted in an increase in size of the cellulose nanostructures. Concerning the effect of the chemical nature of the coagulation bath, water–ethanol mixtures were found to result in no discernible differences in nanostructure, and water–acetone mixtures resulted in a substantial structural transition at very low water fractions. These variations can result in dense or open gel-like morphologies, with the porosity in open morphologies tuneable through bath temperature and water–acetone proportion. A major implication of this is that solvent exchanges after coagulation may still affect structure, as the cellulose sites occupied by strong H-bonders in a wet film could be replaced with a weak H-bonder. There need not be a well-defined porous structure that is formed and fixed upon coagulation that simply undergoes shrinking when drying, but rather the structure can still fundamentally change and be changed after coagulation. This means studies of dry films cannot be reliably used to draw conclusions about wet film structure. However, this also suggests many opportunities towards the engineering of films for both direct use and as precursors of carbon molecular sieve membranes, since the reproducible variation in morphologies means that the internal structure available of the film, for example for the introduction of additives or molecular spacers,^50^ is a tuneable parameter. The success of SANS in characterisation of wet cellulose films suggests that studies of the larger-scale structure seen in acetone- and high-temperature films with ultra-small-angle scattering methods such as spin-echo small-angle scattering (SESANS) would be viable.

## Author contributions

Lassi V. Tiihonen: investigation, data analysis, writing – original draft. Gabriel Bernardo: conceptualization, investigation, writing – original draft, writing – review & editing. Robert Dalgliesh: resources, writing – review & editing. Adélio Mendes: funding acquisition, resources, writing – review & editing. Steven R. Parnell: conceptualization, funding acquisition, data analysis, writing – review & editing.

## Conflicts of interest

There are no conflicts to declare.

## Supplementary Material

RA-014-D4RA00971A-s001
