# Supplementary material for: Influence of the coagulation bath on the nanostructure of cellulose films regenerated from an ionic liquid solution
Source: RSC Adv. 2024 Apr 22;14(18):12888–96. doi: 10.1039/d4ra00971a (PMC11033612; doi:10.1039/d4ra00971a)
Supplement: RA-014-D4RA00971A-s001 [file RA-014-D4RA00971A-s001.pdf]

Electronic Supplementary Material (ESI) for RSC Advances.
This journal is © The Royal Society of Chemistry 2024

# Supplementary Information: Influence of the coagulation bath on the nanostructure of cellulose films regenerated from an ionic liquid solution

[a] Faculty of Applied Sciences, Delft University of Technology, 2629 JB, Delft, Netherlands.
[b] LEPABE - Laboratory for Process Engineering, Environment, Biotechnology and Energy, Faculty of Engineering, University of Porto, Rua Dr. Roberto Frias, 4200-465 Porto, Portugal.
[c] ALiCE - Associate Laboratory in Chemical Engineering, Faculty of Engineering, University of Porto, Rua Dr. Roberto Frias, 4200-465 Porto, Portugal.
[d] ISIS pulsed Neutron and Muon Source, Rutherford Appleton Laboratory, Chilton, Oxfordshire, OX11 0QX , United Kingdom
† Corresponding e-mails: gbernardo@fe.up.pt; S.R.Parnell@tudelft.nl
Electronic Supplementary Information (ESI) available: [details of any supplementary information available should be included here]. See DOI: 10.1039/x0xx00000x

Lassi V. Tiihonen,[a] Gabriel Bernardo*[b,c] , Robert Dalgliesh[d], Adélio Mendes[b,c], and Steven R. Parnell*[a]

# S1 – FULL DATA AND FIT PLOTS

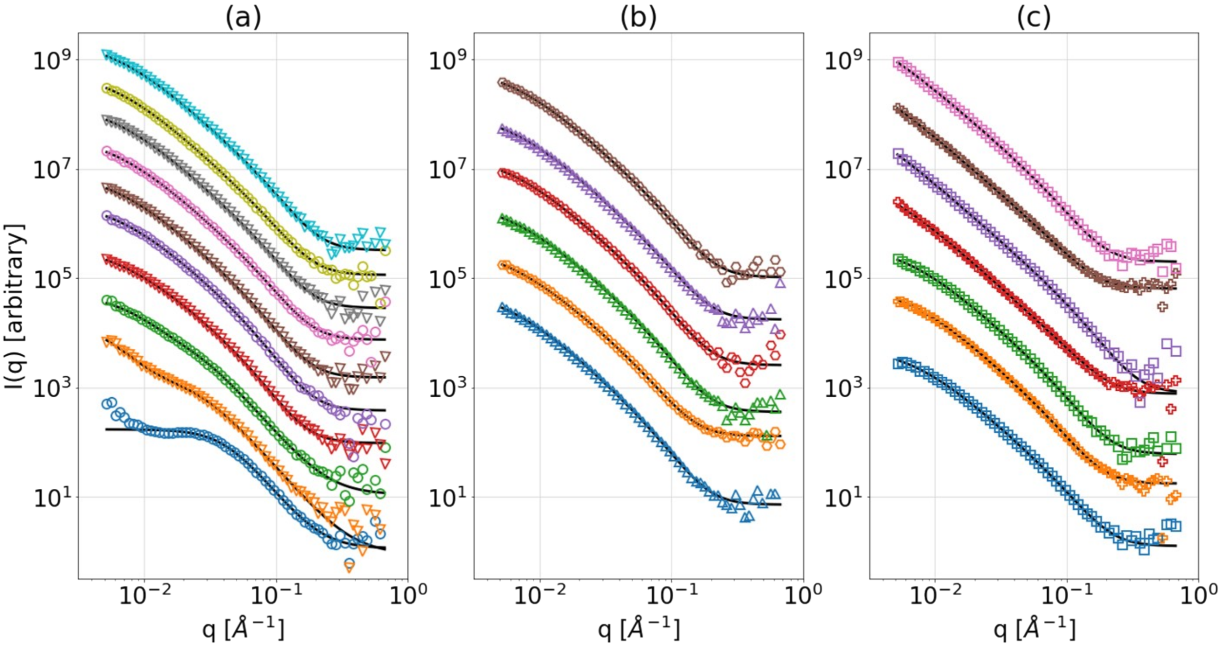


**(a) From bottom to top: 100:0, 99:1, 98:2, 95:5, 90:10, 80:20, 60:40, 40:60, 20:80, 0:100 acetone:$H_2O$**

**(b) From bottom to top: 100:0, 80:20, 60:40, 40:60, 20:80, 0:100 ethanol:$H_2O$**

**(c) From bottom to top: 5, 20 (pale), 20, 40, 60, 80, 80(reproduced) °C**

S2 – IMAGES

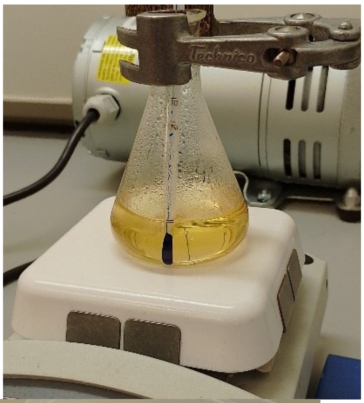

Pale solution

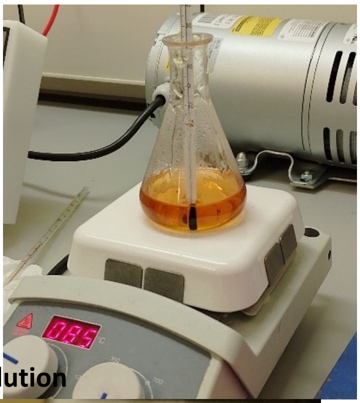

Fully browned solution

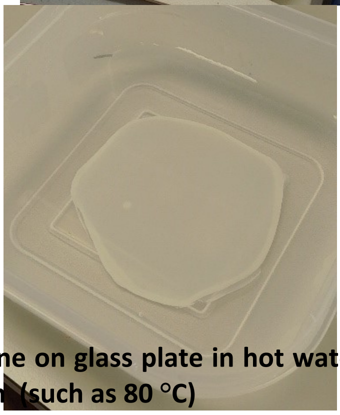

Cloudy membrane on glass plate in hot water coagulation bath (such as 80 °C)

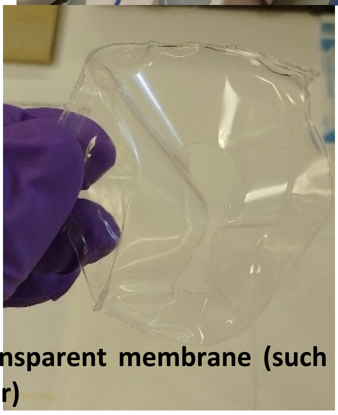

Dried highly transparent membrane (such as from 100% water)

## S3 – PALE-BROWNED SOLUTION COMPARISON

A "pale" cellulose solution was used to prepare a water-coagulated sample before the fully browned solution. Adjusting for calliper-measured sample thickness resulted in identical SANS data within uncertainties.

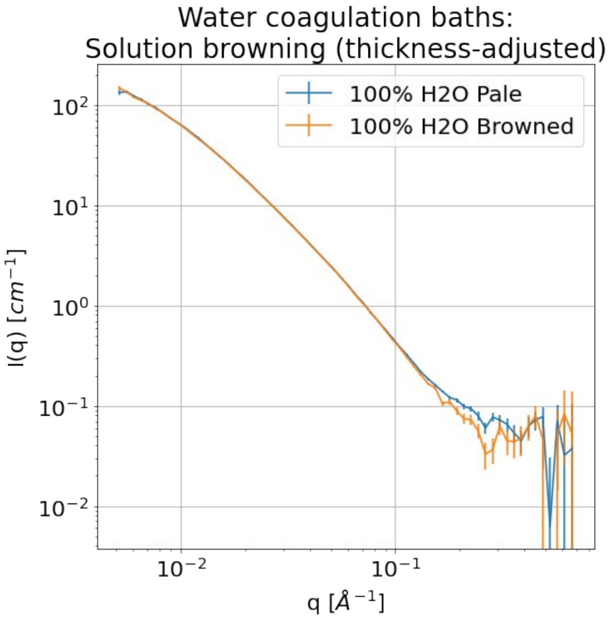

# S4 – METHANOL-ETHANOL COMPARISON

Pure methanol and ethanol resulted in highly similar SANS results. Subtraction of the estimated excess high-q background from the methanol signal shows the data are nearly identical.

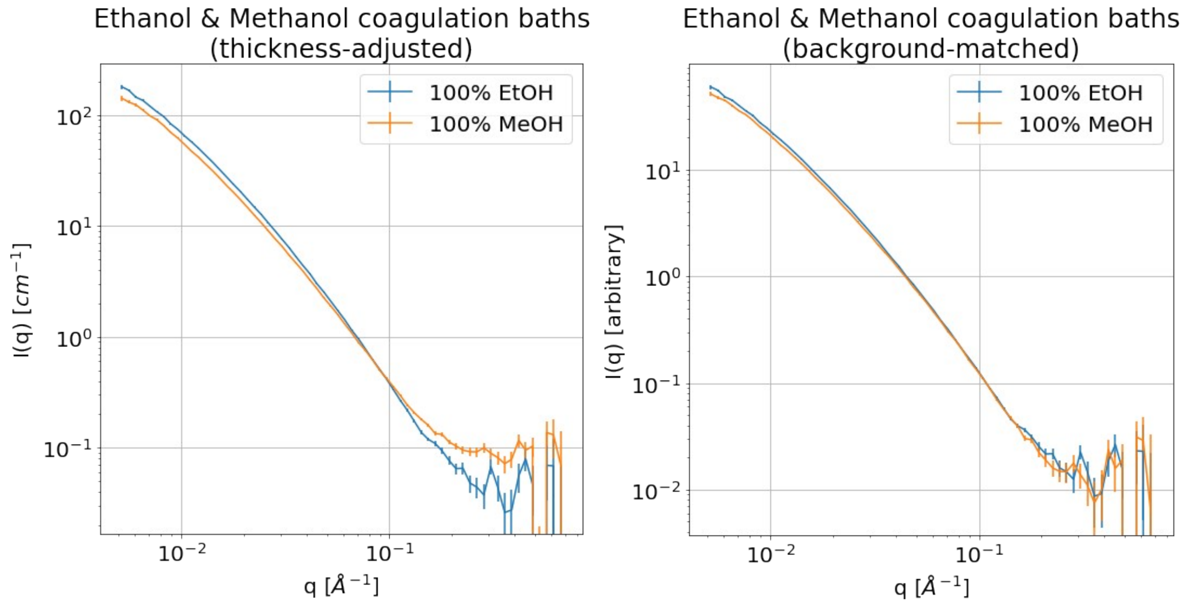


# S5 – REPRODUCTION: HIGH-TEMPERATURE WATER

Reproduction of a high-temperature water sample shows the high reproducibility of SANS results for water coagulation baths.

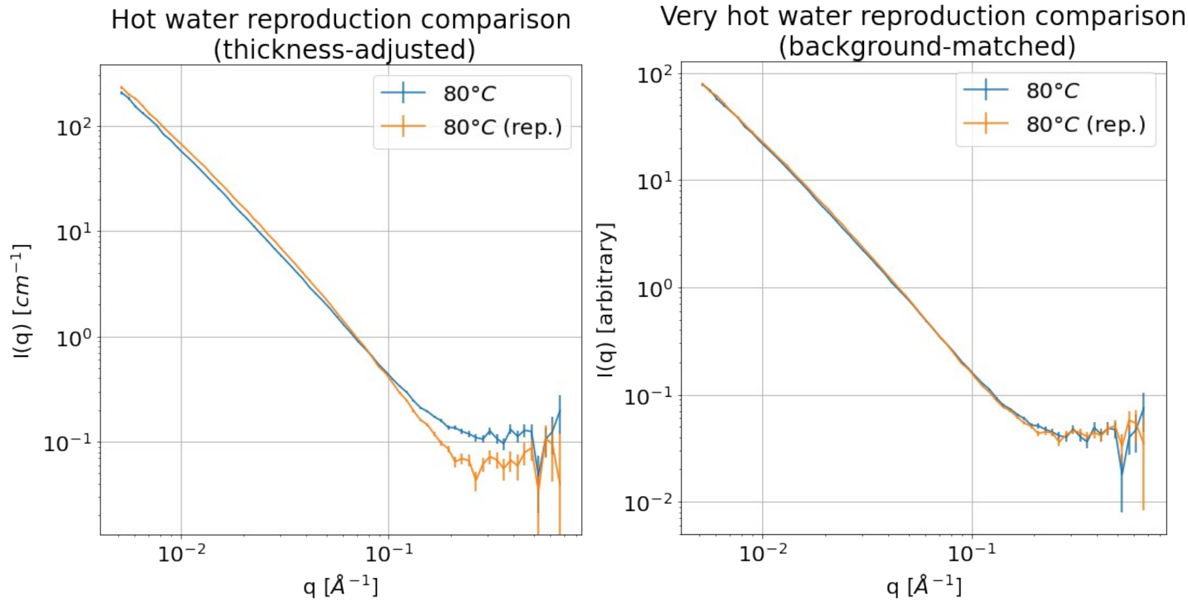

## S6 – MICROBALANCE RESULTS: ACETONE-WATER

Mass fractions as measured by microbalance method for acetone-water samples. The standard deviations were corrected based on the control chart constant $c_4$ for unbiased uncertainty estimates. *: for the single-sample cases, the highest adjacent standard deviation was assumed.

| **vol% H2O** | $mean(\phi_{m,cel})$ | $N_{samples}$ | $STD(\phi_{m,cel})$ | $c_4(N)$ | $STD_{corr}(\phi)$ |
|---|---|---|---|---|---|
| **0** | 18.56 | 9 | 1.56 | 0.97 | 1.61 |
| **1** | 22.07 | 2 | 0.81 | 0.80 | 1.02 |
| **2** | 20.49 | 3 | 0.46 | 0.89 | 0.52 |
| **5** | 18.04 | 4 | 1.36 | 0.92 | 1.48 |
| **10** | 13.37 | 3 | 0.68 | 0.89 | 0.76 |
| **20** | 13.01 | 3 | 0.20 | 0.89 | 0.23 |
| **30** | 12.46 | 1 | 0.28* | - | 0.31* |
| **40** | 12.25 | 1 | 0.28* | - | 0.31* |
| **50** | 12.32 | 3 | 0.28 | 0.89 | 0.31 |
| **100** | 12.02 | 2 | 0.12 | 0.80 | 0.15 |

## S7 – MICROBALANCE RESULTS: WATER TEMPERATURE

Mass fractions as measured by microbalance method for water temperature samples. The standard deviations were corrected based on the control chart constant $c_4$ for unbiased uncertainty estimates. *: for the single-sample cases, the highest adjacent standard deviation was assumed.

| **H2O temp** | $mean(\phi_{m,cel})$ | $N_{samples}$ | $STD(\phi_{m,cel})$ | $c_4(N)$ | $STD_{corr}(\phi)$ |
|---|---|---|---|---|---|
| **5** | 11.4 | 1 | 0.17* | - | 0.21* |
| **20** | 11.3 | 2 | 0.17 | 0.80 | 0.21 |
| **40** | 9.97 | 4 | 0.34 | 0.92 | 0.37 |
| **60** | 8.62 | 3 | 0.33 | 0.89 | 0.37 |
| **80** | 8.55 | 3 | 0.19 | 0.89 | 0.21 |

## S8 – SASVIEW FITTING SAMPLE LEGEND & THICKNESSES:

| **Label** | **Description** | **Thickness [mm]** |
|---|---|---|
| Wet01 | Pale solution - Room temp water (lab room temp 19.7 C) | 0.403 |
| Wet02 | Browned solution - Room temp water (lab room temp 19.7 C) | 0.310 |
| Wet03 | " Ice water (~5 C) | 0.267 |
| Wet04 | " Hot water (~60 C) | 0.350 |
| Wet05 | " Very hot water (~80 C) | 0.377 |
| Wet06 | " Warm water (~40 C) | 0.340 |
| Wet07 | " Very hot water (~80 C) (reproduced with shorter delay) | 0.337 |
| Wet08 | 100% acetone | 0.184 |
| Wet09 | 80:20% acetone:H2O | 0.283 |
| Wet10 | 60:40% acetone:H2O | 0.317 |
| Wet11 | 40:60% acetone:H2O | 0.283 |
| Wet12 | 20:80% acetone:H2O | 0.277 |

| | | |
|---|---|---|
| Wet13 | 100% H2O | 0.283 |
| Wet14 | 90:10% acetone:H2O | 0.330 |
| Wet15 | 95:5% acetone:H2O | 0.227 |
| Wet16 | 98:2% acetone:H2O | 0.187 |
| Wet17 | 100% ethanol | 0.333 |
| Wet18 | 100% methanol | 0.363 |
| Wet19 | 80:20% ethanol:H2O | 0.333 |
| Wet20 | 60:40% ethanol:H2O | 0.313 |
| Wet21 | 40:60% ethanol:H2O | 0.300 |
| Wet22 | 20:80% ethanol:H2O | 0.283 |
| Wet23 | 99:1% acetone:H2O | 0.117 |
| Wet24 | 99.5:0.5% acetone:H2O | 0.117 |

# S9 – SASVIEW FITTING OUTPUTS: ACETONE-WATER

## PMF fitting outputs (gray = fixed values):

| | scale | scale_err | background | background_err | xi1 | xi1_err | xi2 | xi2_err | d1 | d2 | d2_err | sld_polymer | sld_nonsolvent |
|---|---|---|---|---|---|---|---|---|---|---|---|---|---|
| **Wet02** | 0.040147 | 0.001394 | 0.012366 | 0.00082 | 8.8269 | 0.24773 | 170.8 | 4.0327 | 2 | 2.0722 | 0.010147 | 1.75 | 6.4 |
| **Wet08** | 0.048441 | 8.66 | 0.018946 | 0.001873 | -16.23 | 1485.7 | 16.224 | 1091.4 | 2 | 2.3578 | 5.8942 | 1.75 | 6.4 |
| **Wet09** | 0.040347 | 0.001795 | 0.014846 | 0.000719 | 10.956 | 0.29993 | 170.32 | 4.5817 | 2 | 2.0657 | 0.012695 | 1.75 | 6.4 |
| **Wet10** | 0.04496 | 0.001665 | 0.018231 | 0.000773 | 10.005 | 0.25376 | 176.3 | 4.356 | 2 | 2.0645 | 0.010688 | 1.75 | 6.4 |
| **Wet11** | 0.041577 | 0.001508 | 0.017327 | 0.00078 | 9.6572 | 0.25366 | 174.87 | 4.2367 | 2 | 2.0758 | 0.010485 | 1.75 | 6.4 |
| **Wet12** | 0.035553 | 0.00131 | 0.017487 | 0.000987 | 8.5683 | 0.2829 | 175.38 | 4.4343 | 2 | 2.0962 | 0.010689 | 1.75 | 6.4 |
| **Wet14** | 0.060817 | 0.002934 | 0.01477 | 0.000826 | 12.337 | 0.31262 | 171.49 | 4.7554 | 2 | 2.0249 | 0.013643 | 1.75 | 6.4 |
| **Wet15** | 0.059418 | 0.004135 | 0.014954 | 0.000647 | 14.01 | 0.42469 | 169.27 | 6.3575 | 2 | 1.9431 | 0.019604 | 1.75 | 6.4 |
| **Wet16** | 0.26403 | 0.044644 | 0.01253 | 0.000618 | 20.896 | 0.98862 | 544.5 | 178.01 | 2 | 1.4434 | 0.048539 | 1.75 | 6.4 |
| **Wet23** | 0.71495 | 0.076519 | 0.001058 | 0.000876 | 23.293 | 0.84381 | 2.08E+08 | 1.00E+08 | 2 | 1.05 | 0.027291 | 1.75 | 6.4 |

## DL fitting outputs (gray = fixed values):

| | scale | background | background_err | A_scale | A_scale_err | A_cor_length | A_cor_length_err | B_lorentz_scale_1 | B_lorentz_scale_1_err | B_lorentz_length_1 | B_lorentz_length_1_err | B_lorentz_exp_1 | B_lorentz_exp_1_err |
|---|---|---|---|---|---|---|---|---|---|---|---|---|---|
| **Wet 02** | 1 | 0.003598 | 0.001006 | 6.77E-05 | 1.80E-06 | 93.839 | 1.3212 | 3.7711 | 0.19925 | 36.635 | 0.9566 | 2.6224 | 0.021607 |
| **Wet 08** | 1 | 0.011362 | 0.00085 | 0 | 0 | 0 | 0 | 1.6933 | 0.01004 | 24.279 | 0.11087 | 3.0266 | 0.018871 |
| **Wet 09** | 1 | 0.006024 | 0.000911 | 5.99E-05 | 1.85E-06 | 98.588 | 1.6218 | 4.2451 | 0.23575 | 39.421 | 1.0038 | 2.7085 | 0.020326 |
| **Wet 10** | 1 | 0.008508 | 0.000968 | 6.94E-05 | 1.85E-06 | 97.866 | 1.4143 | 4.5036 | 0.22621 | 38.304 | 0.91428 | 2.6703 | 0.019334 |
| **Wet 11** | 1 | 0.007414 | 0.000964 | 6.51E-05 | 1.88E-06 | 9.96E+01 | 1.5413 | 4.6404 | 0.24819 | 39.65 | 1.0084 | 2.6376 | 0.019064 |
| **Wet 12** | 1 | 0.006778 | 0.001167 | 5.67E-05 | 2.16E-06 | 103.88 | 2.054 | 5.0557 | 0.35613 | 43.306 | 1.4804 | 2.5353 | 0.021177 |
| **Wet 14** | 1 | 0.005642 | 0.001072 | 8.31E-05 | 2.06E-06 | 93 | 1.23 | 4.562 | 0.2097 | 36.007 | 0.75165 | 2.8194 | 0.021037 |
| **Wet 15** | 1 | 0.007865 | 0.000877 | 5.53E-05 | 1.86E-06 | 90.631 | 1.5432 | 3.4869 | 0.18565 | 36.424 | 0.8566 | 2.8325 | 0.022904 |
| **Wet 16** | 1 | 0.006896 | 0.00087 | 2.88E-05 | 1.30E-06 | 100.79 | 2.5148 | 3.5273 | 0.16427 | 37.968 | 0.83739 | 2.7698 | 0.022017 |
| **Wet 23** | 1 | 0.002269 | 0.00124 | 9.15E-06 | 4.55E-07 | 174.57 | 11.959 | 3.1749 | 0.11528 | 37.291 | 0.80422 | 2.7387 | 0.031133 |

# S10 – SASVIEW FITTING OUTPUTS: ETHANOL-WATER

## PMF fitting outputs (gray = fixed values):

| | scale | scale_err | background | background_err | xi1 | xi1_err | xi2 | xi2_err | d1 | d2 | d2_err | sld_polymer | sld_nonsolvent |
|---|---|---|---|---|---|---|---|---|---|---|---|---|---|
| **Wet13** | 0.041577 | 0.001508 | 0.017327 | 0.00078 | 9.6572 | 0.25366 | 174.87 | 4.2367 | 2 | 2.0758 | 0.010485 | 1.75 | 6.4 |
| **Wet17** | 0.042117 | 0.001522 | 0.014374 | 0.000732 | 10.549 | 0.25385 | 188.36 | 4.7451 | 2 | 2.0923 | 0.010308 | 1.75 | 6.4 |
| **Wet19** | 0.054 | 0.00228 | 0.036835 | 0.000753 | 11.388 | 0.27628 | 182.4 | 5.0578 | 2 | 2.027 | 0.01207 | 1.75 | 6.4 |
| **Wet20** | 0.051413 | 0.00201 | 0.014505 | 0.000737 | 10.884 | 0.25861 | 179.68 | 4.6359 | 2 | 2.0431 | 0.011219 | 1.75 | 6.4 |
| **Wet21** | 0.047649 | 0.001665 | 0.014888 | 0.000768 | 10.002 | 0.23925 | 178.26 | 4.2204 | 2 | 2.0656 | 0.010089 | 1.75 | 6.4 |
| **Wet22** | 0.032548 | 0.001115 | 0.014568 | 0.000823 | 8.5376 | 0.2596 | 169.87 | 3.8966 | 2 | 2.118 | 0.00995 | 1.75 | 6.4 |

## DL fitting outputs:

| | background | background_err | A_scale | A_scale_err | A_cor_length | A_cor_length_err | B_lorentz_scale_1 | B_lorentz_scale_1_err | B_lorentz_length_1 | B_lorentz_length_1_err | B_lorentz_exp_1 | B_lorentz_exp_1_err |
|---|---|---|---|---|---|---|---|---|---|---|---|---|
| **Wet 02** | 0.0035979 | 0.001006 | 6.77E-05 | 1.80E-06 | 93.839 | 1.3212 | 3.7711 | 0.19925 | 36.635 | 0.9566 | 2.6224 | 0.021607 |
| **Wet 17** | 0.0028949 | 0.000902 | 6.25E-05 | 1.92E-06 | 111.41 | 1.9468 | 6.3686 | 0.34132 | 44.622 | 1.085 | 2.6518 | 0.015686 |
| **Wet 19** | 0.02709 | 0.000971 | 7.19E-05 | 1.85E-06 | 97.252 | 1.3681 | 4.5934 | 0.21478 | 37.593 | 0.81823 | 2.7364 | 0.019293 |
| **Wet 20** | 0.005283 | 0.000943 | 7.56E-05 | 1.80E-06 | 95.811 | 1.2533 | 4.3705 | 0.19726 | 36.666 | 0.77665 | 2.738 | 0.019301 |
| **Wet 21** | 0.0048075 | 0.00096 | 7.44E-05 | 1.84E-06 | 9.83E+01 | 1.3291 | 4.749 | 0.22255 | 38.108 | 0.84912 | 2.6777 | 0.018315 |
| **Wet 22** | 0.0036039 | 0.000952 | 4.97E-05 | 2.19E-06 | 110.82 | 2.537 | 6.1786 | 0.44873 | 47.907 | 1.6163 | 2.5232 | 0.016708 |

# S11 – SASVIEW FITTING OUTPUTS: WATER TEMPERATURE

## PMF fitting outputs (gray = fixed values):

| | scale | scale_err | background | background_err | xi1 | xi1_err | xi2 | xi2_err | d1 | d2 | d2_err | sld_polymer | sld_nonsolvent |
|---|---|---|---|---|---|---|---|---|---|---|---|---|---|
| **Wet01** | 0.051643 | 0.001582 | 0.024979 | 0.000843 | 9.0888 | 0.21313 | 168.52 | 3.4381 | 2 | 2.078 | 0.008943 | 1.75 | 6.4 |
| **Wet02** | 0.040147 | 0.0013938 | 0.012366 | 0.00082 | 8.8269 | 0.24773 | 170.8 | 4.0327 | 2 | 2.0722 | 0.010147 | 1.75 | 6.4 |
| **Wet03** | 0.032985 | 0.0012266 | 0.012528 | 0.000889 | 7.7342 | 0.27767 | 153.09 | 3.5775 | 2 | 2.0719 | 0.011019 | 1.75 | 6.4 |
| **Wet04** | 0.034335 | 0.00071407 | 0.003347 | 0.001018 | 5.9689 | 0.21176 | 437.09 | 28.3 | 2 | 2.0748 | 0.006195 | 1.75 | 6.4 |
| **Wet05** | 0.038188 | 0.00095696 | 0.038378 | 0.000931 | 7.6277 | 0.21572 | 425.88 | 28.823 | 2 | 2.056 | 0.007365 | 1.75 | 6.4 |
| **Wet06** | 0.029757 | 0.00065676 | 0.022349 | 0.0011 | 5.5827 | 0.23885 | 268.48 | 8.5408 | 2 | 2.1191 | 0.006562 | 1.75 | 6.4 |
| **Wet07** | 0.043974 | 0.0012562 | 0.016988 | 0.000795 | 9.3094 | 0.21649 | 382.85 | 22.809 | 2 | 2.0362 | 0.008306 | 1.75 | 6.4 |

## DL fitting outputs:

| | Chi2 | background | background_err | A_scale | A_scale_err | A_cor_length | A_cor_length_err | B_lorentz_scale_1 | B_lorentz_scale_1_err | B_lorentz_length_1 | B_lorentz_length_1_err | B_lorentz_exp_1 | B_lorentz_exp_1_err |
|---|---|---|---|---|---|---|---|---|---|---|---|---|---|
| **Wet 01** | 2.5274 | 0.01504 | 0.001044 | 9.37E-05 | 1.93E-06 | 91.341 | 1.0169 | 4.3947 | 0.18754 | 34.727 | 0.72599 | 2.6812 | 0.019192 |
| **Wet 02** | 2.6275 | 0.0035979 | 0.001006 | 6.77E-05 | 1.80E-06 | 93.839 | 1.3212 | 3.7711 | 0.19925 | 36.635 | 0.9566 | 2.6224 | 0.021607 |
| **Wet 03** | 2.1737 | 0.0061326 | 0.001065 | 6.11E-05 | 1.71E-06 | 84.776 | 1.1919 | 2.4986 | 0.14745 | 32.974 | 1.0064 | 2.6009 | 0.028561 |
| **Wet 04** | 3.5694 | -0.0082309 | 0.001051 | 5.05E-05 | 1.36E-06 | 139.01 | 2.6754 | 6.7488 | 0.43935 | 52.412 | 1.8467 | 2.317 | 0.016208 |
| **Wet 05** | 3.6776 | 0.025104 | 0.001041 | 4.73E-05 | 1.39E-06 | 1.47E+02 | 3.2459 | 7.9435 | 0.49822 | 54.574 | 1.7688 | 2.3872 | 0.015138 |
| **Wet 06** | 3.1287 | 0.0071824 | 0.000864 | 1.31E-05 | 1.14E-06 | 409.85 | 192.07 | 54.796 | 12.573 | 141.62 | 14.873 | 2.2326 | 0.0066 |
| **Wet 07** | 4.4302 | 0.0034785 | 0.000945 | 4.94E-05 | 1.34E-06 | 144.05 | 2.9642 | 7.9746 | 0.42262 | 51.989 | 1.3716 | 2.4862 | 0.014182 |
